# Diagnostic value of miR-92a in asymptomatic carotid artery stenosis patients and its ability to predict cerebrovascular events

**DOI:** 10.1186/s13000-020-00987-z

**Published:** 2020-06-10

**Authors:** Gang Chen, Jianwei Gao, Yuguo Sheng, Xinqiang Han, Xingang Ji, Mengpeng Zhao, Jian Wu

**Affiliations:** Department of Vascular Interventional, Binzhou Medical University Hospital, Binzhou, 256603 China

**Keywords:** Asymptomatic carotid artery stenosis, microRNA-92a, Diagnosis, Cerebrovascular events

## Abstract

**Background:**

Early diagnosis of asymptomatic carotid artery stenosis (ACAS) is important to prevent the incidence of cerebrovascular events. This study aimed to investigate the circulating expression of microRNA-92a (miR-92a) in ACAS patients and evaluate its diagnostic value for ACAS and predictive value for cerebrovascular events.

**Methods:**

Circulating expression of miR-92a was measured using quantitative real-time PCR. Chi-square test was used to analyze the association of miR-92a with ACAS patients’ clinical characteristics. A receiver operating characteristic (ROC) was used to evaluate the diagnostic value of miR-92a, and the Kaplan-Meier method and Cox regression analysis were used to assess the predictive value of miR-92a for cerebrovascular events.

**Results:**

Serum expression of miR-92a was higher in ACAS patients than that in the healthy controls (*P* <  0.001), and associated with patients’ degree of carotid stenosis (*P* = 0.013). The elevated miR-92a expression could distinguish ACAS patients from healthy individual, and was an independent predictive factor for the occurrence of cerebrovascular events (*P* = 0.015).

**Conclusion:**

The data from this study indicated that circulating increased miR-92a may serve as a noninvasive diagnostic biomarker for ACAS and a potential risk factor for the future onset of cerebrovascular events.

## Introduction

Stroke, a major cerebrovascular event, is a severe healthcare burden worldwide. According to the statistics, stroke ranks the second leading cause of global deaths, inferior to ischemic heart disease [1]. Carotid artery stenosis (CAS) is the main risk of cerebrovascular events, and estimated to cause about 20–25% of all strokes [2]. CAS can be divided into symptomatic CAS (SCAS) and asymptomatic CAS (ACAS) according to the clinical manifestation. SCAS refers to the patients who have a history of transient ischemic attack (TIA) or stoke, while ACAS is defined as the patients with luminal carotid narrowing but have no history of stroke or other cerebrovascular events in the previous 6 months [3]. Currently, carotid endarterectomy (CEA) and carotid artery stenting (CAS) are two efficient therapeutic strategies for SCAS patients [4]. However, the delayed diagnosis of ACAS, which due to the lack of clinical symptoms, leads to the occurrence of cerebrovascular events. Therefore, novel strategies for diagnosis and cerebrovascular event prediction are urgently needed for patients with ACAS.

Emerging studies have reported the important role of microRNAs (miRNAs) in various human diseases [5]. miRNAs are a group of small noncoding RNAs with regulatory effect on gene expression at the post-transcriptional level [6]. By binding to the 3′-untranslated region of target mRNAs, the expression of key genes that serve pivotal roles in various pathogenesis can be inhibited by miRNAs [7]. Thus, miRNAs can be involved in the regulation of various cellular processes, such as the cell proliferation, migration and apoptosis of vascular endothelial cells and vascular smooth muscle cells, which are closely related to vascular remodeling and the development of CAS [8, 9]. Some miRNAs with aberrant expression patterns have been identified in patients with CAS and are involved in the disease progression [10, 11], providing potential insight into the methods to improve CAS diagnosis and management.

MiR-17-92 cluster plays a crucial role in the physiological and pathological processes of CAS [12]. Several members of the miR-17-92 cluster are upregulated in carotid arteries exhibiting restenosis after angioplasty and stenting when compared to the adjacent normal artery, including miR-17, miR-18a, miR-19a, miR-20a, and miR-92a [13]. A recent study reported by Huang et al. has proposed that hypertensive patients with a high miR-92a serum level had a high risk of atherosclerosis, and the elevated circulating miR-92a might serve as a novel diagnostic biomarker for atherosclerosis, which is a major cause of CAS [14]. In addition, the increased expression of miR-92a has also been found in patients with diabetes [15] and hyperlipidemia [16], which represent two common risk factors of CAS. Moreover, several target genes of miR-92a have been determined to be involved in the regulation of atherosclerosis and CAS, such as phosphatase and tensin homolog (PTEN), SMAD family member 7 (SMAD7) and fibroblast growth factor receptor substrate 2 (FRS2) [17–19]. However, the expression and clinical role of miR-92a in CAS has rarely been reported. This study was carried out to investigate the circulating expression of miR-92a in ACAS patients, and evaluate the diagnostic performance of miR-92a for CAS screening and the predictive value of miR-92a for the occurrence of cerebrovascular events.

## Materials and methods

### Patients and serum collection

This study was approved by the Ethics Committee of Binzhou Medical University Hospital, and the written consent was obtained from each participant before blood sampling. A total of 122 ACAS patients were enrolled in this study from Binzhou Medical University Hospital between 2011 and 2013 following the inclusion criteria: (1) more than 18 years old; (2) the stenotic degree of ipsilateral internal carotid artery more than 50%; (3) no history of TIA or stroke; (4) no malignancies. A color Doppler ultrasound was used to diagnose CAS with a high-resolution duplex ultrasound system and a probe at scanner frequency of 7–10 MHz. The ultrasonography examination was performed by two experienced radiologists and the degree of stenosis was calculated. Besides, 62 healthy volunteers were recruited in this study as normal controls, who had no history of metabolic diseases, malignancies, cardiovascular diseases, cerebrovascular diseases, or inflammatory diseases. Venous blood was collected from the participants and used to isolate serum samples using centrifugation. The demographic and clinicopathological characteristics of the patients were recorded for further analysis.

### Follow-up

All the patients were recruited in a 5-year follow-up survey. The occurrence of ipsilateral cerebrovascular events, including stroke, TIA, or sudden death, was defined as the primary endpoint. The patients who died from other unrelated incidents were excluded from the analysis. The diagnosis of stroke was determined by the combination of clinical symptom and brain imaging examination, including Doppler ultrasound, MRI, CT, and angiography [20]. The TIA cases were those with clinical symptoms disappear within 24 h.

### RNA extraction

Total RNA in the collected serum was extracted using TRIzol reagent (Invitrogen, Carlsbad, CA, USA) following the manufacturer’s instruction. The concentration and purity of RNA were determined by a NanoDrop 2000 (Thermo Fisher Scientific, Waltham, MA, USA). The pure RNA was used for reverse transcription to synthesize cDNA using a PrimeScript RT reagent kit (TaKaRa, Shiga, Japan) according to the protocols of the manufacturer.

### Quantitative real-time PCR (qRT-PCR)

The expression of miR-92a in serum samples was estimated using qRT-PCR, which was carried out using a SYBR green I Master Mix kit (Invitrogen, Carlsbad, CA, USA) and the 7500 Real-Time PCR System (Applied Biosystems, USA). U6 was used as an internal control in the reactions. The relative expression value of miR-92a was calculated by using the 2^−ΔΔCt^ method and normalized to U6. The primers used were as follows: miR-92a forward, 5′-TATTGCACTTGTCCCGGCCTGT-3′ and reverse, 5′- CTTTCTACACAGGTTGGGATCG-3′; and U6 forward, 5′-CTCGCTTCGGCAGCACA-3′ and reverse, 5′-AACGCTTCACGAATTTGCGT-3′.

### Statistical analysis

The data obtained in this study were expressed as mean ± standard deviation (SD) and analyzed using SPSS 21.0 software (SPSS Inc., Chicago, IL) and GraphPad Prism 7.0 software (GraphPad Software, Inc., USA). Differences between groups were analyzed using student’s *t* test. Chi-square test was used to estimate the relationship between miR-92a and patients’ clinical data. A receiver operating characteristic (ROC) curve was constructed to evaluate the diagnostic value of circulating miR-92a, and the area under the curve (AUC) was calculated to examine the diagnostic accuracy. The value of miR-92a on predicting cerebrovascular diseases was analyzed using the Kaplan-Meier method and Cox regression analysis. A *P* value of less than 0.05 was considered statistically significant.

## Results

### Clinical data comparisons between healthy control and ACAS patients groups

The demographic and clinicopathological characteristics of the control groups and ACAS patients group were summarized in Table 1. It was found that there was no significant difference for age, gender, body mass index (BMI), and high-density lipoprotein cholesterol (HDL-C) between the control and ACAS group (all *P* > 0.05). However, the levels of fasting blood glucose (*P* <  0.001), total cholesterol (TC, *P* = 0.001) and low-density lipoprotein (LDL-C, *P* <  0.001) were significantly higher in ACAS group than that in the control group, and more cases with hypertension were identified in ACAS group (*P* <  0.001).

**Table 1:** Clinical data comparisons between healthy control and ACAS patients groups

Parameters	Control (*n* = 62)	Asymptomatic CAS (*n* = 122)	*P* values
Age (years), mean ± SD	58.89 ± 7.37	60.34 ± 7.86	0.226
Gender (Female/male)	18/44	46/76	0.243
BMI (kg/m^2^), mean ± SD	25.37 ± 4.57	26.02 ± 5.28	0.415
Fasting blood glucose (mmol/L), mean ± SD	4.98 ± 0.61	5.86 ± 1.07	< 0.001
Hypertension, n (%)	15 (24.19)	75 (61.48)	< 0.001
TC (mmol/L), mean ± SD	4.74 ± 0.48	5.13 ± 0.79	0.001
LDL-C (mmol/L), mean ± SD	2.71 ± 0.35	3.02 ± 0.49	< 0.001
HDL-C (mmol/L), mean ± SD	1.11 ± 0.15	1.07 ± 0.27	0.217

*BMI* Body mass index, *TC* total cholesterol, *LDL-C*, low density lipoprotein, *HDL-C* high density lipoprotein cholesterol

### Circulating expression of miR-92a in patients with ACAS

**Fig. 1:**
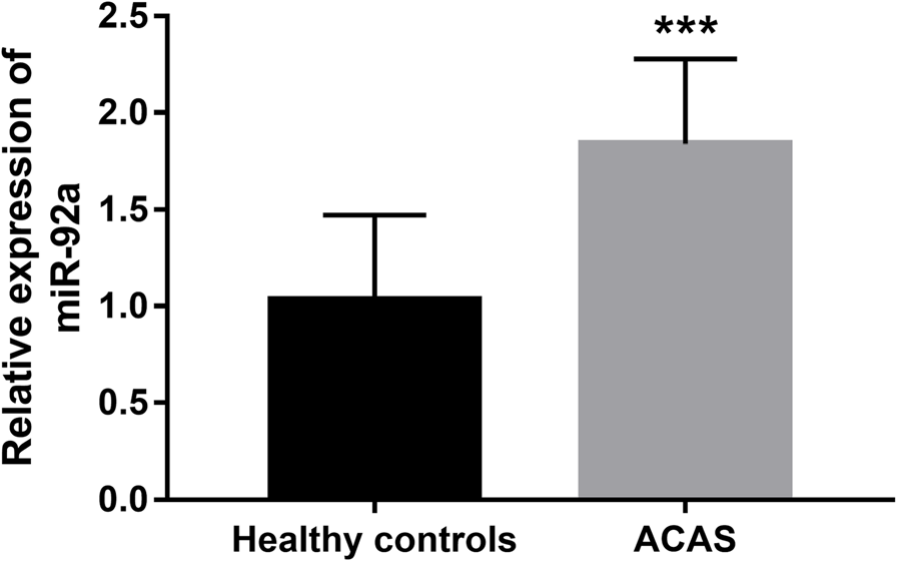
Circulating expression of miR-92a detected by qRT-PCR in patients with ACAS. ***P < 0.001

According to qRT-PCR, the expression of miR-92a in serum samples was significantly higher in patients with ACAS than that in the healthy volunteers, and the difference reached statistical difference (*P* < 0.001, Fig. 1).

### Association of miR-92a with the clinicopathological features of ACAS patients

The demographic and clinicopathological characteristics of ACAS patients were recorded and summarized in Table 2. To understand the role of the dysregulated miR-92a in the development of ACAS, its relationship with the patients’ clinical data was assessed. To facilitate the analysis, all ACAS patients were grouped into low expression and high expression groups according to the mean expression value of miR-92a. As shown in Table 2, the levels of fasting blood glucose (*P* = 0.040), TC (*P* = 0.046) were significantly high in high miR-92a expression group than that in low miR-92a expression group, and more patients with hypertension (*P* = 0.035) and high degree of carotid stenosis (*P* = 0.013) were observed in high miR-92a group. The results indicated that the increased miR-92a expression was associated with fasting blood glucose, TC, hypertension, and degree of carotid stenosis. No significant relationship was detected between miR-92a and age, gender, BMI, LDL-C, or HDL-C (all *P* > 0.05).

**Table 2:** Association of miR-92a with the clinical data of ACSA patients

Parameters	Low miR-92a expression group (*n* = 58)	High miR-92a expression group (*n* = 64)	*P* values
Age (years), mean ± SD	60.43 ± 7.76	60.27 ± 8.01	0.908
Gender (Female/male)	22/36	24/40	0.961
BMI (kg/m^2^), mean ± SD	25.69 ± 5.30	26.32 ± 5.27	0.513
Fasting blood glucose (mmol/L), mean ± SD	5.65 ± 1.00	6.05 ± 1.10	0.040
Hypertension, n (%)	30 (40.00)	45 (60.00)	0.035
TC (mmol/L), mean ± SD	4.98 ± 0.80	5.26 ± 0.78	0.046
LDL-C (mmol/L), mean ± SD	2.98 ± 0.52	3.06 ± 0.47	0.377
HDL-C (mmol/L), mean ± SD	1.09 ± 0.24	1.05 ± 0.30	0.520
Degree of carotid stenosis (≥ 70%), n (%)	28 (38.36)	45 (61.64)	0.013

*BMI* Body mass index, *TC* total cholesterol, *LDL-C* low density lipoprotein, *HDL-C* high density lipoprotein cholesterol

### Diagnostic value of miR-92a in patients with ACAS

**Fig. 2:**
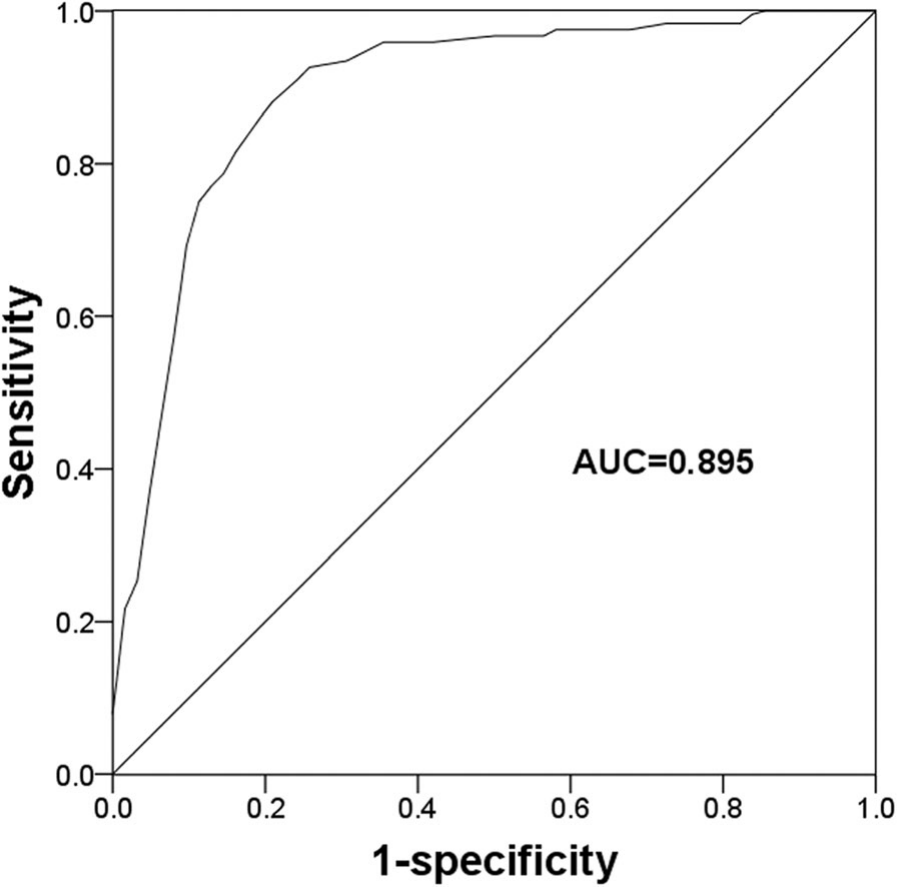
A ROC curve constructed based on serum miR-92a in ACAS patients and healthy individuals. AUC, area under the curve

According to the serum levels of miR-92a in ACAS patients, this study further evaluated the diagnostic significance of miR-92a for ACAS by constructing a ROC curve (Fig. 2). Based on the serum miR-92a levels, the AUC in the ROC analysis was 0.895, indicating the relatively high diagnostic accuracy of miR-92a in differentiation between ACAS patients and healthy individuals. The optimal cutoff value was 1.285, with a sensitivity of 88.5% and a specificity of 79.0%.

### Predictive value of miR-92a for the occurrence of cerebrovascular diseases

**Fig. 3:**
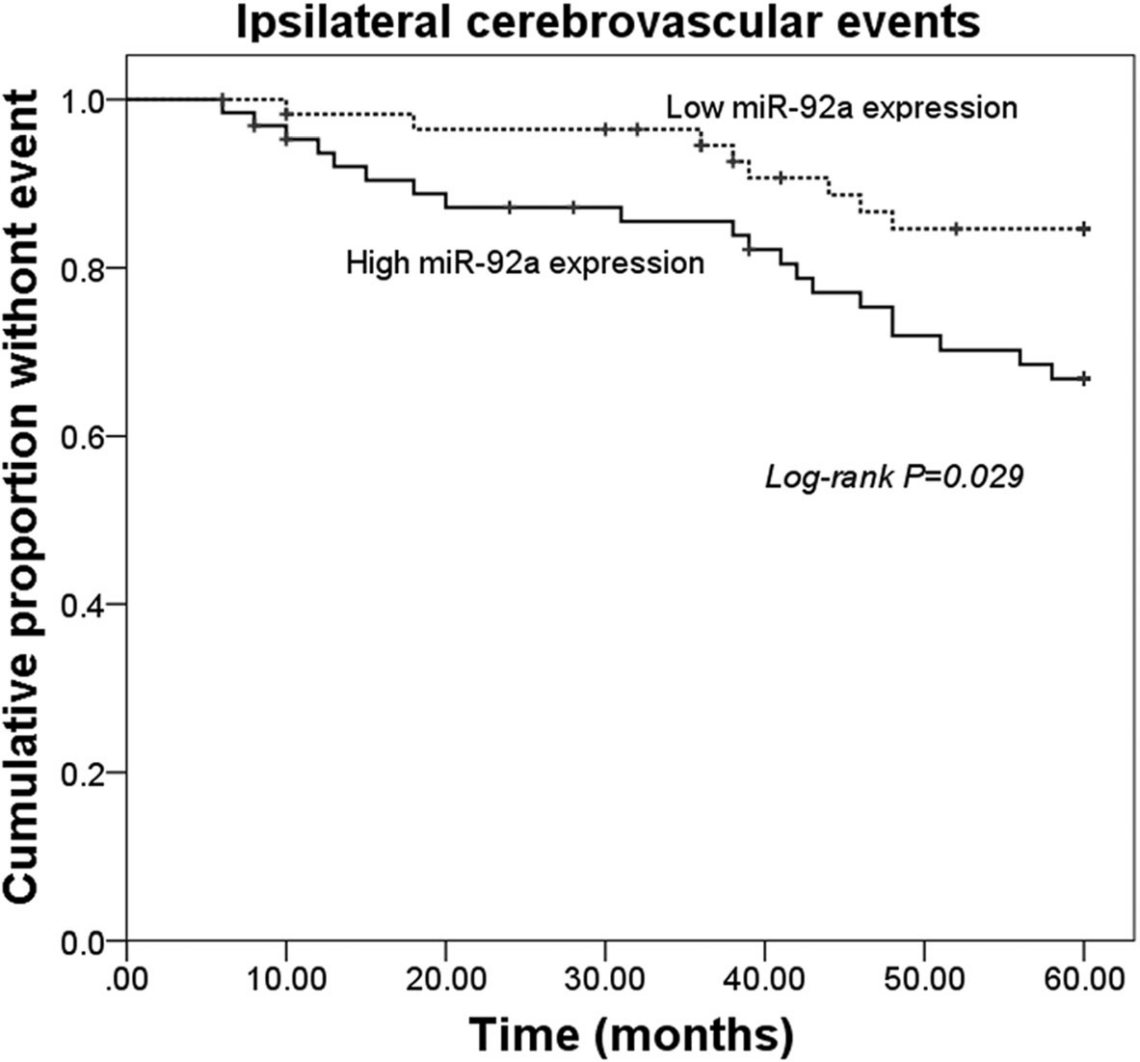
Kaplan-Meier survival curves for ACAS patients with different expression levels of miR-92a. Log-rank *P* = 0.029

According to the 5-year follow-up survey, cerebrovascular events occurred in 28 patients, including 20 TIA and 8 strokes. Among 28 patients who suffered from cerebrovascular events, 8 cases were observed in the low miR-92a expression group, while 20 cases were in the high miR-92a expression group. No death nor contralateral ischemic events occurred. By a Kaplan-Meier analysis, the patients with high miR-92a expression experienced more cerebrovascular events compared with those with low miR-92a expression (log-rank *P* = 0.029, Fig. 3). The further determine the variables that might predict the occurrence of cerebrovascular events, all risk factors were included in a Cox regression analysis. As shown in Table 3, the univariate data indicated that more cerebrovascular events occurred in patients with higher miR-92a (HR = 4.121, 95% CI = 1.366–8.496, *P* = 0.008) and larger degree of carotid stenosis (HR = 2.668, 95% CI =1.189–5.992, *P* = 0.022). The subsequent multivariate analysis data revealed that ACAS patients with higher miR-92a (HR = 2.971, 95% CI = 1.230–7.173, *P* = 0.015) or larger degree of carotid stenosis (HR = 2.515, 95% CI =1.103–5.738, *P* = 0.028) had high risk for the occurrence of cerebrovascular events.

**Table 3:** Cox regression analysis in patients with ACSA

Parameters	Univariate analysis		Multiple analysis	
	HR (95% CI)	*P* value	HR (95% CI)	*P* value
miR-92a	4.121 (1.366–8.496)	0.008	2.971 (1.230–7.173)	0.015
Age	1.521 (0.725–3.192)	0.268	1.402 (0.618–3.181)	0.419
Gender	1.025 (0.480–2.189)	0.949	0.921 (0.409–2.078)	0.844
BMI	1.920 (0.845–4.360)	0.119	0.450 (0.191–1.065)	0.069
Fasting blood glucose	1.172 (0.530–2.453)	0.755	0.932 (0.408–2.126)	0.866
Hypertension	1.233 (0.584–2.505)	0.734	1.223 (0.541–2.769)	0.629
TC	1.100 (0.520–2.325)	0.803	0.974 (0.382–2.483)	0.956
LDL-C	1.281 (0.610–2.693)	0.513	1.233 (0.453–3.358)	0.682
HDL-C	1.258 (0.599–2.645)	0.544	1.169 (0.467–2.928)	0.739
Degree of carotid stenosis	2.668 (1.189–5.992)	0.022	2.515 (1.103–5.738)	0.028

*BMI* Body mass index, *TC* total cholesterol, *LDL-C* low density lipoprotein, *HDL-C* high density lipoprotein cholesterol

## Discussion

This study aimed to evaluate the diagnostic performance of miR-92a for ACAS and its predictive value for cerebrovascular events in patients with ACAS by analyzing miR-92a expression levels. According to qRT-PCR, circulating miR-92a expression was elevated in ACAS patients compared with healthy individuals. The serum increased miR-92a expression levels were associated with the degree of carotid stenosis and some vascular risk factors in ACAS patients. According to the deregulated expression pattern, the diagnostic value of miR-92a was evaluated, and the ROC data demonstrated the relatively high diagnostic accuracy of serum miR-92a for ACAS patients. Furthermore, the occurrence of cerebrovascular disease was recorded by a 5-year follow-up survey, and we found that ACAS patients with high miR-92a experienced more cerebrovascular events, suggesting that miR-92a might serve as a risk factor for the occurrence of cerebrovascular event.

CAS is a progressive pathological condition, which is generally considered as a result of atherosclerosis and plaque rupture [21]. The prevention and therapy of CAS, especially the ACAS, have attracted increasing attention for their significant promoting effects on the occurrence of cerebrovascular events, such as TIA and stroke [22]. Over the past decades, a large number of miRNAs are involved in the regulation of some pathophysiological cellular processes and functional signaling pathway, which contributes to the development of various human diseases [23]. The endothelial cell biological function, lipid homeostasis, mechanosensing, and leukocyte recruitment, which represent important cellular processes during atherosclerosis, have been reported to be regulated by miRNAs [24–26]. A study reported by Zheng et al. demonstrated that miR-155 could be involved in the progression of atherosclerosis by regulating endothelial cell proliferation and migration, and exosome-mediated transfer of miR-155 might serve as a novel therapeutic target for atherosclerosis [27]. Another study reported by Gong et al. investigated the role of miR-150 in atherosclerosis rats and found that miR-150 ablation could ameliorate atherosclerosis and improve plaque stability through regulating smooth muscle cell function, macrophage infiltration and lipid accumulation [28]. These published studies implied the critical regulatory role of miRNAs in the progression of atherosclerosis and plaque stability. Of note, some miRNAs involved in the progression of atherosclerosis were also differentially expressed in the pathogenesis of CAS and might participate in disease development [29]. For example, miR-145, which has been reported to be related to atherosclerosis progression, was reduced in patients with CAS and suppressed disease progression by inhibiting vascular smooth muscle cell proliferation [11, 30]. The increased miR-330-5p was found in unstable carotid plaques, which contributes to the progression of CAS and was proposed as a target for atherosclerotic disease therapy [31].

MiR-92a has been determined as one of the vascular-related microRNAs [32], and can be involved in the development of vascular-associated diseases by regulating vascular formation and remodeling [33]. The elevation in miR-92a expression has been reported in patients with atherosclerosis, and was identified as a noninvasive atherosclerosis biomarker in hypertensive patients [14]. Wiese et al. recently provided evidence for miR-92a to regulate the development of renal injury-associated atherosclerosis [34]. This study firstly investigated the expression patterns of miR-92a in patients with ACAS and found that serum miR-92a expression was significantly increased in ACAS patients compared with healthy controls, which was consistent with the expression data in atherosclerosis patients [14]. The relationship analysis data revealed that serum miR-92a expression was associated with patients’ carotid stenosis, indicating that miR-92a might be involved in the development of CAS. Moreover, the association of miR-92a with the vascular risk factors, including diabetes, hypertension, and dyslipidemia was also found in the present study. A recent study proposed that the deregulated miR-92a in diabetes mellitus had a promoting effect on the development of cardiovascular disease by enhancing NF-κB signaling and inflammatory response [15]. Niculescu et al. found that the inhibition of miR-92a might be a novel method to decrease liver and plasma cholesterol levels in animals with dyslipidemia [35]. Additionally, it is well known that the dysregulation of miR-92a functions in the progression of diseases by regulating the targeting genes. Regarding the association between miR-92a and arteriosclerosis, Huang et al. have elucidated the mechanism in rats. They found that inhibiting miR-92a protects endothelial progenitor cells by targeting GDF11 via SMAD2/3/FAK/Akt/eNOS pathway [36]. Wang et al. demonstrated that miR-92a is elevated in coronary atherosclerosis patients, and the ATP binding cassette A1(ABCA1) is determined to be the target gene [37]. These previous study results combined with the relationship analysis data in our study led us to conclude that miR-92a might be involved in the development of CAS through promoting the progress vascular risk events. However, further studies are needed to explore the underlying mechanism of the involvement of miR-92a in CAS. Additionally, in the present study, only ACAS patients were included, further studies are needed to determine the expression changes of miR-92a in CAS patients for the comparison to the healthy controls and ACAS group.

Serum miRNAs are generally considered a group of good diagnostic tools for various pathological conditions and diseases [38]. The dysregulation of circulating miR-92a has been determined as a diagnostic biomarker for patients with atherosclerosis [14], acute myeloid leukemia [39], and cervical cancer [40]. In this study, a ROC curve based on serum miR-92a was constructed and suggested that miR-92a could serve as a promising diagnostic biomarker to screen ACAS patients. Early diagnosis of CAS, especially for ACAS patients who had no significant clinical manifestations, is very important for the prevention of cardiovascular and cerebrovascular diseases. Stroke and TIA are the most common cerebrovascular diseases processed from CAS, and stroke remains a fatal disease threatening human life and health. In a study reported by He et al., miR-92a was suggested to serve as a regulator of the onset of depression in patients with stroke [41], indicating that miR-92a is associated with stroke progression. Thus, this study further assessed the relationship between miR-92a expression and the onset of cerebrovascular events. The incidence rate of cerebrovascular events in our research cohort was 23.0%, and the patients with high miR-92a experienced more TIE and stroke cases. The Cox regression data demonstrated that miR-92a could independently predict the occurrence of TIE and stroke, suggesting that miR-92a serves as a risk factor in ACAS patients to predict future cerebrovascular events. Moreover, it should be noted that a close association of miR-92a with various malignancies, such as colorectal and gastric cancers, has been detected [42, 43]. Therefore, in the present study, cases with no history of malignancies were included to avoid the influence of malignancies on serum miR-92a expression. Moreover, in the future studies about the role of miR-92a in CAS, the influence of malignant tumors should be taken into account.

In conclusion, elevated miR-92a expression was detected in ACAS patients, which is associated with carotid stenosis and serves as a candidate diagnostic biomarker for ACAS. MiR-92a has the potential as a predictor for the onset of cerebrovascular events in ACAS patients. Although our results provide a novel insight into the value of miR-92a in ACAS diagnosis and future cerebrovascular events prediction, further investigations with a larger study population are needed to confirm the role of miR-92a.

## Data Availability

The datasets used and/or analyzed during the current study are available from the corresponding author on reasonable request.

## Abbreviations

ACAS: Asymptomatic carotid artery stenosis
miR-92a: microRNA-92a
CAS: Carotid artery stenosis
SCAS: Symptomatic CAS
TIA: Transient ischemic attack
CEA: Carotid endarterectomy
ROC: Receiver operating characteristic
AUC: Area under the curve
BMI: Body mass index
PTEN: Phosphatase and tensin homolog
SMAD7: SMAD family member 7
FRS2: fibroblast growth factor receptor substrate 2

## References

[1] Mortimer R, Nachiappan S, Howlett DC (2018). Carotid artery stenosis screening: where are we now?. Br J Radiol.

[2] Dharmakidari S, Bhattacharya P, Chaturvedi S (2017). Carotid artery stenosis: medical therapy, surgery, and stenting. Curr Neurol Neurosci Reports.

[3] Halliday A, Mansfield A, Marro J, Peto C, Peto R, Potter J (2004). Prevention of disabling and fatal strokes by successful carotid endarterectomy in patients without recent neurological symptoms: randomised controlled trial. Lancet (London, England).

[4] Yoshida K, Miyamoto S (2015). Evidence for management of carotid artery stenosis. Neurol Med Chir.

[5] Volný O, Kašičková L, Coufalová D, Cimflová P, Novák J (2015). microRNAs in Cerebrovascular Disease. Adv Exp Med Biol.

[6] Xu F, Zhou F (2019). Inhibition of microRNA-92a ameliorates lipopolysaccharide-induced endothelial barrier dysfunction by targeting ITGA5 through the PI3K/Akt signaling pathway in human pulmonary microvascular endothelial cells. Int Immunopharmacol.

[7] Wei Q, Tu Y, Zuo L, Zhao J, Chang Z, Zou Y (2019). MiR-345-3p attenuates apoptosis and inflammation caused by oxidized low-density lipoprotein by targeting TRAF6 via TAK1/p38/NF-kB signaling in endothelial cells. Life Sci.

[8] Khoo CP, Roubelakis MG, Schrader JB, Tsaknakis G, Konietzny R, Kessler B (2017). miR-193a-3p interaction with HMGB1 downregulates human endothelial cell proliferation and migration. Sci Report.

[9] Zhang R, Sui L, Hong X, Yang M, Li W (2017). MiR-448 promotes vascular smooth muscle cell proliferation and migration in through directly targeting MEF2C. Environ Sci Pollut Res Int.

[10] Dolz S, Górriz D, Tembl JI, Sánchez D, Fortea G, Parkhutik V (2017). Circulating MicroRNAs as novel biomarkers of stenosis progression in asymptomatic carotid stenosis. Stroke..

[11] Han Z, Hu H, Yin M, Li X, Li J, Liu L (2018). miR-145 is critical for modulation of vascular smooth muscle cell proliferation in human carotid artery stenosis. J Biol Regul Homeost Agents.

[12] Luo T, Cui S, Bian C, Yu X (2014). Crosstalk between TGF-beta/Smad3 and BMP/BMPR2 signaling pathways via miR-17-92 cluster in carotid artery restenosis. Mol Cell Biochem.

[13] Johnson JL (2019). Elucidating the contributory role of microRNA to cardiovascular diseases (a review). Vasc Pharmacol.

[14] Huang Y, Tang S, Ji-Yan C, Huang C, Li J, Cai AP (2017). Circulating miR-92a expression level in patients with essential hypertension: a potential marker of atherosclerosis. J Hum Hypertens.

[15] Wang WY, Zheng YS, Li ZG, Cui YM, Jiang JC (2019). MiR-92a contributes to the cardiovascular disease development in diabetes mellitus through NF-κB and downstream inflammatory pathways. Eur Rev Med Pharmacol Sci.

[16] Loh WP, Yang Y, Lam KP (2017). miR-92a enhances recombinant protein productivity in CHO cells by increasing intracellular cholesterol levels. Biotechnol J.

[17] Moulton KS, Li M, Strand K, Burgett S, McClatchey P, Tucker R (2018). PTEN deficiency promotes pathological vascular remodeling of human coronary arteries. JCI Insight.

[18] Wei L, Zhao S, Wang G, Zhang S, Luo W, Qin Z (2018). SMAD7 methylation as a novel marker in atherosclerosis. Biochem Biophys Res Commun.

[19] Huang T, Zhao HY, Zhang XB, Gao XL, Peng WP, Zhou Y (2020). LncRNA ANRIL regulates cell proliferation and migration via sponging miR-339-5p and regulating FRS2 expression in atherosclerosis. Eur Rev Med Pharmacol Sci.

[20] Rudd AG, Bowen A, Young GR, James MA (2017). The latest national clinical guideline for stroke. Clin Med (London, England).

[21] Meschia JF, Klaas JP, Brown RD, Brott TG (2017). Evaluation and Management of Atherosclerotic Carotid Stenosis. Mayo Clin Proc.

[22] Liu S, Cai J, Ge F, Yue W (2017). The risk of ischemic events increased in patients with asymptomatic carotid stenosis with decreased cerebrovascular reserve. J Investig Med.

[23] Hajibabaie F, Kouhpayeh S, Mirian M, Rahimmanesh I, Boshtam M, Sadeghian L (2019). MicroRNAs as the actors in the atherosclerosis scenario. J Physiol Biochem.

[24] Gimbrone MA, García-Cardeña G (2016). Endothelial cell dysfunction and the pathobiology of atherosclerosis. Circ Res.

[25] Singh V, Rana M, Jain M, Singh N, Naqvi A, Malasoni R (2015). Curcuma oil attenuates accelerated atherosclerosis and macrophage foam-cell formation by modulating genes involved in plaque stability, lipid homeostasis and inflammation. Br J Nutr.

[26] Merino H, Parthasarathy S, Singla DK (2013). Partial ligation-induced carotid artery occlusion induces leukocyte recruitment and lipid accumulation--a shear stress model of atherosclerosis. Mol Cell Biochem.

[27] Zheng B, Yin WN, Suzuki T, Zhang XH, Zhang Y, Song LL (2017). Exosome-mediated miR-155 transfer from smooth muscle cells to endothelial cells induces endothelial injury and promotes atherosclerosis. Mol Ther.

[28] Gong FH, Cheng WL, Wang H, Gao M, Qin JJ, Zhang Y (2018). Reduced atherosclerosis lesion size, inflammatory response in miR-150 knockout mice via macrophage effects. J Lipid Res.

[29] Eken SM, Jin H, Chernogubova E, Li Y, Simon N, Sun C (2017). MicroRNA-210 enhances fibrous cap stability in advanced atherosclerotic lesions. Circ Res.

[30] Liu K, Xuekelati S, Zhang Y, Yin Y, Li Y, Chai R (2017). Expression levels of atherosclerosis-associated miR-143 and miR-145 in the plasma of patients with hyperhomocysteinaemia. BMC Cardiovasc Disord.

[31] Wei X, Sun Y, Han T, Zhu J, Xie Y, Wang S (2019). Upregulation of miR-330-5p is associated with carotid plaque's stability by targeting Talin-1 in symptomatic carotid stenosis patients. BMC Cardiovasc Disord.

[32] Hijmans JG, Diehl KJ, Bammert TD, Kavlich PJ, Lincenberg GM, Greiner JJ (2018). Association between hypertension and circulating vascular-related microRNAs. J Hum Hypertens.

[33] Zhang L, Zhou M, Wang Y, Huang W, Qin G, Weintraub NL (2014). miR-92a inhibits vascular smooth muscle cell apoptosis: role of the MKK4-JNK pathway. Apoptosis.

[34] Wiese CB, Zhong J, Xu ZQ, Zhang Y, Ramirez Solano MA, Zhu W (2019). Dual inhibition of endothelial miR-92a-3p and miR-489-3p reduces renal injury-associated atherosclerosis. Atherosclerosis.

[35] Niculescu LS, Simionescu N, Fuior EV, Stancu CS, Carnuta MG, Dulceanu MD (2018). Inhibition of miR-486 and miR-92a decreases liver and plasma cholesterol levels by modulating lipid-related genes in hyperlipidemic hamsters. Mol Biol Rep.

[36] Huang HT, Liu ZC, Wu KQ, Gu SR, Lu TC, Zhong CJ (2019). MiR-92a regulates endothelial progenitor cells (EPCs) by targeting GDF11 via activate SMAD2/3/FAK/Akt/eNOS pathway. Ann Trans Med.

[37] Wang Z, Zhang J, Zhang S, Yan S, Wang Z, Wang C (2019). MiR30e and miR92a are related to atherosclerosis by targeting ABCA1. Mol Med Rep.

[38] Mirzaei H, Momeni F, Saadatpour L, Sahebkar A, Goodarzi M, Masoudifar A (2018). MicroRNA: relevance to stroke diagnosis, prognosis, and therapy. J Cell Physiol.

[39] Elhamamsy AR, El Sharkawy MS, Zanaty AF, Mahrous MA, Mohamed AE, Abushaaban EA (2017). Circulating miR-92a, miR-143 and miR-342 in plasma are novel potential biomarkers for acute myeloid leukemia. Int J Mol Cell Med.

[40] Kong Q, Tang Z, Xiang F, Jiang J, Yue H, Wu R (2017). Diagnostic value of serum hsa-mir-92a in patients with cervical Cancer. Clin Lab.

[41] He JR, Zhang Y, Lu WJ, Liang HB, Tu XQ, Ma FY (2017). Age-Related Frontal Periventricular White Matter Hyperintensities and miR-92a-3p Are Associated with Early-Onset Post-Stroke Depression. Front Aging Neurosci.

[42] Chen E, Li Q, Wang H, Yang F, Min L, Yang J (2018). MiR-92a promotes tumorigenesis of colorectal cancer, a transcriptomic and functional based study. Biomed Pharmacother.

[43] Tao XC, Zhang XY, Sun SB, Wu DQ (2019). miR92a contributes to cell proliferation, apoptosis and doxorubicin chemosensitivity in gastric carcinoma cells. Oncol Rep.

